# First-Trimester Urine Concentrations of Phthalate Metabolites and Phenols and Placenta miRNA Expression in a Cohort of U.S. Women

**DOI:** 10.1289/ehp.1408409

**Published:** 2015-06-19

**Authors:** Jessica LaRocca, Alexandra M. Binder, Thomas F. McElrath, Karin B. Michels

**Affiliations:** 1 Harvard University Center for the Environment, Harvard University, Cambridge, Massachusetts, USA; 2 Obstetrics and Gynecology Epidemiology Center, Department of Obstetrics, Gynecology, and Reproductive Biology, Brigham and Women’s Hospital, Harvard Medical School, Boston, Massachusetts, USA; 3 Department of Epidemiology, Harvard T.H. Chan School of Public Health, Boston, Massachusetts, USA; 4 Division of Maternal–Fetal Medicine, Brigham and Women’s Hospital, Boston, Massachusetts, USA

## Abstract

**Background:**

There is increasing concern that early-life exposure to endocrine-disrupting chemicals (EDCs) can influence the risk of disease development. Phthalates and phenols are two classes of suspected EDCs that are used in a variety of everyday consumer products, including plastics, epoxy resins, and cosmetics. *In utero* exposure to EDCs may affect disease propensity through epigenetic mechanisms.

**Objective:**

The objective of this study was to determine whether prenatal exposure to multiple EDCs is associated with changes in miRNA expression of human placenta, and whether miRNA alterations are associated with birth outcomes.

**Methods:**

Our study was restricted to a total of 179 women co-enrolled in the Harvard Epigenetic Birth Cohort and the Predictors of Preeclampsia Study. We analyzed associations between first-trimester urine concentrations of 8 phenols and 11 phthalate metabolites and expression of 29 candidate miRNAs in placenta by qRT-PCR.

**Results:**

For three miRNAs—miR-142-3p, miR15a-5p, and miR-185—we detected associations between Σphthalates or Σphenols on expression levels (*p* < 0.05). By assessing gene ontology enrichment, we determined the potential mRNA targets of these microRNAs predicted *in silico* were associated with several biological pathways, including the regulation of protein serine/threonine kinase activity. Four gene ontology biological processes were enriched among genes significantly correlated with the expression of miRNAs associated with EDC burden.

**Conclusions:**

Overall, these results suggest that prenatal phenol and phthalate exposure is associated with altered miRNA expression in placenta, suggesting a potential mechanism of EDC toxicity in humans.

**Citation:**

LaRocca J, Binder AM, McElrath TF, Michels KB. 2016. First-trimester urine concentrations of phthalate metabolites and phenols and placenta miRNA expression in a cohort of U.S. women. Environ Health Perspect 124:380–387; http://dx.doi.org/10.1289/ehp.1408409

## Introduction

Prior epidemiologic studies have indicated that unfavorable gestational conditions or exposures such as preeclampsia, maternal obesity, gestational diabetes, alcohol consumption, and smoking can influence epigenetic profiles in placenta and potentially impact risk for adverse health outcomes (Blair et al. 2013; Lesseur et al. 2014; Maccani et al. 2010; Wilhelm-Benartzi et al. 2012). There is increasing concern that exposure to certain environmental pollutants can influence the risk of disease. Endocrine-disrupting chemicals (EDCs) are of concern because they can antagonize or mimic the effects of the body’s endogenous hormones, such as testosterone, estrogen, or thyroid hormone (Birnbaum 2013). Prenatal exposure to EDCs is particularly worrisome because chemical exposure of a hormonally sensitive organ in early life can result in phenotypic organizational changes that may persist throughout life (Colborn et al. 1993). Phenols and phthalates are two classes of suspected EDCs that have recently been of particular concern partly because of their widespread use in consumer products and high production volumes. Several population-based studies have described the exposure profiles of phenols and phthalates in the United States, including specific vulnerable subgroups such as pregnant women (Braun et al. 2011; Chevrier et al. 2013; Meeker et al. 2013; Swan 2008; Wolff et al. 2008; Woodruff et al. 2011).

There is accumulating evidence that epigenetic mechanisms play an important role in mediating the impact of environmental exposures on disease risk. Epigenetics is the study of mitotically heritable and stable modifications in the regulation of gene expression that occur without changes to the underlying DNA sequence. Epigenetic mechanisms include DNA methylation, histone modification, and noncoding RNAs (such as microRNAs). MicroRNAs (miRNAs) are noncoding RNAs that are approximately 22 nucleotides in length. In animals, miRNAs can regulate gene expression post-transcriptionally by imperfect complementarity with a target mRNA, thereby inhibiting protein synthesis (Ambros 2004). Concentrations of phthalate metabolites in urine were associated with the expression of target genes related to trophoblast differentiation and steroidogenesis in placentas collected from 54 women at delivery (Adibi et al. 2010), and miRNA expression in two placenta cell lines was altered following exposure to BPA *in vitro* (Avissar-Whiting et al. 2010). Several miRNAs have been associated with placental health and function, as well as pregnancy disorders (Morales-Prieto et al. 2014).

To date, few studies have examined epigenetic profiles in tissues following exposure to phenols or phthalates, and most were performed using animal models (Bernal and Jirtle 2010; Doshi et al. 2013; Kim et al. 2013; Kundakovic et al. 2013; Susiarjo et al. 2013). *In vivo* and *in vitro* studies have revealed that exposure to bisphenol A (BPA) can alter miRNA expression (Avissar-Whiting et al. 2010; Tilghman et al. 2012; Veiga-Lopez et al. 2013). Prior studies have demonstrated that exposure to BPA and other endocrine-disrupting chemicals interferes with gene expression, providing justification for evaluating changes in the transcriptome (Fletcher et al. 2013; Melzer et al. 2011). There is also emerging evidence that phthalates and phenols may disrupt thyroid function (Boas et al. 2012). However, to our knowledge, no studies to date have examined the relationship between phthalate and phenol exposure to miRNA expression in human tissue. Given that the placenta plays a critical role in fetal growth, and that the first trimester marks a critical period in epigenetic reprogramming and a vulnerable window of exposure, the objective of this study was to determine whether first-trimester exposure to phenols and phthalates may disrupt miRNA expression in the placenta.

## Materials and Methods

*Study population.* Our study population consisted of women concurrently enrolled in two large birth cohorts, the Harvard Epigenetic Birth Cohort (HEBC) and the Predictors of Preeclampsia Study (POPS) at the Brigham and Women’s Hospital in Boston, Massachusetts. Data and biospecimens for the HEBC were collected from June 2007 through June 2009; the cohort includes 1,941 mother–child dyads (Michels et al. 2011). The POPS study is a prospective study of women beginning their prenatal care within clinics and private practices affiliated with the Brigham and Women’s Hospital (*n* = 1,608) in 2007. Urine samples were gathered at the first prenatal visit (< 16 weeks gestation), and at four additional visits during pregnancy. Additional information about this cohort has been published elsewhere (McElrath et al. 2012). Our initial study population consisted of 196 women enrolled in both studies who contributed a first-trimester urine sample between 2007 and 2009. Subsequent miRNA experiments were restricted to mother–infant dyads that had information on infant sex, available placenta for RNA processing, and were not twins, resulting in a final sample size of 179. Study participant characteristics for these 179 women are listed in Table 1, and study participant selection is shown in Supplemental Material, Figure S1.

**Table 1:** Study participant characteristics.

Characteristic	*n* (%)
Ethnicity
White non-Hispanic	126 (70.39)
Hispanic or Latino	25 (14.00)
Asian/Pacific Islander	5 (2.79)
Black/African American	23 (12.85)
Infant sex^*a*^
Female	94 (51.51)
Male	85 (47.49)
Maternal smoking during pregnancy
No	174 (97.21)
Yes	5 (2.79)
Prepregnancy body mass index^*a,b*^	25.45 ± 5.74
Maternal age (years)^*a*^	32.91 ± 5.01
Method of delivery^*b*^ (nmiss = 6)
Spontaneous	36 (20.81)
Induced	27 (15.61)
Cesarean section	110 (63.58)
Maternal complications^*b*^ (nmiss = 34)
Preeclampsia	1 (0.69)
Pregnancy-induced hypertension	7 (4.83)
Gestational diabetes	9 (6.21)
Infections	3 (2.07)
Parity
Nulliparous	64 (36.16)
1	66 (37.29)
2	32 (18.08)
≥ 3	15 (8.47)
Gravidity
0	46 (26.74)
1	44 (25.58)
2	38 (22.09)
≥ 3	44 (25.58)
nmiss, number with missing information. ^***a***^Mean ± SD. ^***b***^Chart-abstracted.	

*Ethics statement.* The participation of human subjects occurred after informed consent was obtained. The study protocols were approved by the Institutional Review Board of Brigham and Women’s Hospital.

*Urine sample collection.* Urine samples from the first prenatal visit (< 16 weeks) were collected in polypropylene urine cups and frozen at –80°C. Before biomarker analysis, samples were defrosted at 4°C overnight. After another round of vortexing, samples were aliquoted to 1.6-mL polypropylene tubes and refrozen at –80°C. For each participant, one tube was shipped on dry ice overnight to the Centers for Disease Control and Prevention (CDC) for measurement of urinary concentrations of phthalate metabolites and phenols. To adjust for urine dilution, specific gravity (SG) was measured at Brigham and Women’s Hospital. SG was measured using a handheld refractomer (Atago, Bellevue, WA), which was calibrated with deionized water before each measurement. We adjusted for urine dilution using SG, rather than urinary creatinine, which is likely altered by stage of pregnancy (Cunningham et al. 2005).

*Urinary phthalate and phenol concentrations.* Urinary concentrations of 8 phenols and 11 phthalate metabolites were measured using the on-line solid phase extraction–high performance liquid chromatography–isotope dilution–tandem mass spectrometry approaches described previously (Silva et al. 2008; Ye et al. 2005). We measured concentrations of total (free plus conjugate) species. Quality control materials, prepared at the CDC with pooled human urine, were analyzed in each batch along with standard, blank, and study samples. The limit of detection (LOD) ranged from 0.2 to 1.2 μg/L for phthalates and from 0.2 to 2.3 μg/L for phenols. Urinary concentrations below the LOD were imputed a value equal to one-half of the LOD (Hornung and Reed 1990). We chose the first trimester as our exposure window because early gestation marks a critical and vulnerable period for the development of epigenetic profiles (Langley-Evans et al. 2012).

*Sample preparation and RNA isolation.* All placenta samples used in this study were taken from the upper layer near the umbilical cord (near upper; NU). Using the mirVANA RNA Isolation Kit (Ambion Inc., Austin, TX), miRNA-containing RNA was isolated according to the manufacturer’s protocol.

*miRNA selection.* We performed a pilot project to identify miRNAs to be differentially expressed across a subset of 48 samples. The 48 samples represented the highest and lowest quintiles of exposure for phthalates and phenols. The pilot study analyzed 86 miRNAs among the 48-sample subset using Qiagen miFinder PCR Arrays (see Supplemental Material, Table S1). We chose to validate candidate miRNAs that were statistically significantly associated with additive phenol and phthalate concentration groups. The miRNAs analyzed in this study are listed in Supplemental Material, Table S2. miRNAs were measured using specially designed Qiagen PCR Arrays, which have extremely high reproducibility, thereby eliminating the need for technical triplicates. Example triplicate reproducibility for the miRNAs assessed in this project is shown in Supplemental Material, Figure S2. For the 4 samples with microRNAs measured in triplicate, between 71% and 84% of the expression measurements had a coefficient of variation (CV) < 1. In one sample, reproducibility was lower (CV > 5) for 2 miRNAs (miR-125b-5p and miR-30a-5p), but the reproducibility of these measurements was strong for all other samples (CV < 1). The relative distribution of phthalates and phenols was similar for both the pilot project and the main study (see Supplemental Material, Figure S3). The range of metabolite values in the pilot captured the range of values measured in the entire study. The reproducibility of miRNA expression between the pilot study and the main study is shown in Supplemental Material, Figure S4, demonstrating consistency. The correlation in expression values measured in the pilot and main study ranged from moderate to strong (*r* = 0.43 to 0.82), with approximately 50% having an *r* ≥ 0.6. Two miRNAs showed poor correlation between the two study stages, miR-128 and miR-155-5p. Additionally, these two miRNAs had two samples that were outliers. The sensitivity to the inclusion of these possibly influential points was assessed in the final models. The full study analyzed 29 miRNAs chosen from the pilot project that were found to be differentially expressed across a subset of 48 samples.

*Real time quantitative reverse transcription polymerase chain reaction (qRT-PCR).* All RNA quality was assessed using the nanodrop ND-1000 (NanoDrop), and all RNA used had passing 260/280 values, which was defined as ≥ 1.8. Using the miScript II RT Kit (Qiagen), 250 ng cDNA was reverse-transcribed from RNA according to the manufacturer’s instructions. The PCR reaction condition was as follows: incubated at 25°C for 10 min, 37°C for 120 min, and 85°C for 5 min. The qRT-PCR was performed using the miScript SYBR® Green PCR Kit (Qiagen) and custom designed miScript PCR arrays (Qiagen) according to manufacturer’s instructions on a Life Technologies 7900HT qPCR machine at the Harvard Medical School ICCB Screening Facility with reverse transcription controls. The qRT-PCR cycling conditions were as follows: 95°C for 15 min, and 40 cycles of 94°C for 15 sec, 55°C for 30 sec, and 70°C for 30 sec. All qRT-PCR data were normalized using the average of two small nucleolar RNAs, SNORD61 and SNORD95. Delta Ct (ΔCt) was defined as the expression difference between the target miRNA and the average of the two normalizing nucleolar RNAs: ΔCt = Ct_normalizing RNA_ – Ct_mirRNA._

*Statistical analysis.* Phthalate body burden was defined by four classifications: Σphthalates (the sum of all phthalates), high molecular weight (HMW), low molecular weight (LMW), and DEHP [di(2-ethylhexyl) phthalate] metabolites. Phenol body burden was defined by three classifications: Σphenols (the sum of all phenols), paraben, and non-paraben. MicroRNAs were modeled as a function of each log-transformed EDC body burden measurement, adjusting for maternal age, maternal ethnicity, and self-reported maternal smoking (yes/no), and infant sex. Effect modification by infant sex was assessed by incorporating an interaction term between EDC level and infant sex into our models. In the case of a significant interaction, determined by a Wald test, the association between EDCs and miRNA levels was reported separately for male and female infants. If infant sex was not a significant modifier, the association was reported for male and female infants together. Although we have previously observed that individual phthalate and phenol biomarker concentrations are significantly correlated with one another in their respective chemical groups, we have also observed that phthalate body burden is not strongly correlated with phenol body burden in first-trimester urines (LaRocca et al. 2014). Phthalates and phenols may differentially affect epigenetic modifications, and the combined effect of these multiple chemical exposures is currently unknown. To investigate potential additive synergism or antagonism, we reanalyzed each adjusted model for miRNA level, including a main effect for both Σphenols and Σphthalates, as well as an interaction term between these summations. For all models, significance was determined using an alpha level of 0.05.

MicroRNA targets were predicted *in silico* and based on correlations with gene expression across the genome. The miRNA target prediction algorithm miRWALK (http://zmf.umm.uni-heidelberg.de/apps/zmf/mirwalk/micrornapredictedtarget.html) was used to predict targets for miRNA of interest (Dweep et al. 2011). Target prediction included a comparative analysis by four other prediction programs, miRanda, miRDB, TargetScan, and RNA22. To be considered a predicted target for further investigation, the target must have appeared in at least four of five prediction programs, and have a TargetScan Total context+ score of < 0 (Grimson et al. 2007; Lewis et al. 2005). These scores rank expected response based on predicted seed-pairing stability, target-site abundance, local A–U content, the location of the site within the 3´UTR, and 3´-supplementary pairing. Gene ontology (GO) enrichment for biological processes associated with at least 10 genes was assessed among the list of all the *in silico* predicted targets of the miRNAs significantly associated with EDC levels. To account for GO topology, the Fisher exact test *p*-values were conditioned on neighboring terms (Alexa et al. 2006, 2010). Additionally, suspected miRNA targets were identified by estimating the Spearman correlation between miRNA levels and expression across the genome for a subset (*n* = 109) of individuals with excellent RNA quality. Genome-wide expression was assessed on the Affymetrix GeneChip® Human Gene 2.0 ST Array at the Microarray Core Facility at the Dana-Farber Cancer Institute in Boston. Signal intensities were processed before analysis using the Affymetrix Expression Consule Software (Affymetrix), which included robust multichip analysis (RMA) background correction, quantile normalization, and gene-level summarization of expression using median polish^73,74^. For each miRNA significantly associated with EDC burden, we assessed the Spearman correlation with all RefSeq genes, using a conservative Bonferroni correction to identify significant correlations accounting for multiple testing (25,642 genes, 3 miRNAs). GO ontology enrichment among the genes correlated with miRNA levels was analyzed similarly to the enrichment among *in silico* predicted targets.

## Results

*Phthalate and phenol measurements.* Characteristics of the study population, including maternal age, ethnicity, smoking, and body mass index of 179 pregnant women–newborn dyads are listed in Table 1. We measured 11 phthalate metabolites and 8 phenols in first trimester urine samples from our study participants. SG-adjusted means, limits of detection (LOD), and percent of the population above the LOD are listed in Tables 2 and 3. Phthalate metabolites and phenols were detected in ≥ 80% of samples, except for MEHP [mono(2-ethylhexyl) phthalate] (68% > LOD) (Table 2) and butyl parraben (BuPB) and triclosan (both with 78% > LOD) (Table 3).

**Table 2:** Phthalate metabolite characteristics and measurements.

Phthalate	Abbreviation	Molecular weight (g/mol)	High/low MW	Parent compound	LOD (μg/L)	GM (μg/L)	GM (mol/L)	% above LOD
Mono(2-ethyl-5-carboxypentyl) phthalate	MECPP	308.33	High	Di(2-ethylhexyl) phthalate (DEHP)	0.6	41.78	133.56	100
Mono(2-ethyl-5-hydroxyhexyl) phthalate	MEHHP	294.34	High	DEHP	0.7	29.87	99.73	98.87
Mono(2-ethyl-5-oxohexyl) phthalate	MEOHP	292.32	High	DEHP	0.7	18.39	61.54	97.74
Mono(2-ethylhexyl) phthalate	MEHP	278.34	High	DEHP	1.2	4.38	15.70	68.36
Mono(3-carboxypropyl) phthalate	MCPP	252.22	High	Dioctyl phthalate (DOP)	0.2	1.40	5.45	84.75
Mono(2,6-dimethyl-6-carboxyhexyl) phthalate	MCOP	322.35	High	Diisononyl phthalate (DiNP)	0.7	6.85	20.08	94.92
Mono(2,7-dimethyl-7-carboxyheptyl) phthalate	MCNP	336.38	High	Diisodecyl phthalate (DiDP)	0.6	2.01	5.82	80.23
Monobenzyl phthalate	MBzP	256.25	High	Benzylbutyl phthalate (BzBP)	0.3	5.34	20.14	97.18
Mono-isobutyl phthalate	MiBP	222.24	Low	Diisobutyl phthalate (DiBP)	0.6	5.74	24.84	94.92
Mono-*n*-butyl phthalate	MnBP	222.23	Low	Dibutyl phthalate (DnBP)	0.6	13.05	57.25	97.18
Monoethyl phthalate	MEP	194.18	Low	Diethyl phthalate (DEP)	0.8	85.00	432.68	100
Abbreviations: GM, geometric mean; LOD, limit of detection; MW, molecular weight. Concentrations < LOD were assigned a value equal to one-half the LOD. Correction factors of 0.66 for MEP and 0.72 for MBzP were applied due to analytic standards of inadequate purity. Summed HMW phthalates include all phthalates listed as “high”; summed LMW phthalates include all phthalates listed as “low”; and summed DEHP metabolites include all phthalates with DEHP listed as the parent compound.								

**Table 3:** Phenol characteristics and measurements.

Phenol	Abbreviation	Molecular weight	Paraben (yes/no)	LOD (μg/L)	GM (μg/L)	GM (mol/L)	% > LOD
2,4-Dichlorophenol	DCP	163.00	No	0.2	0.59	3.59	80.23
2,5-Dichlorophenol	2,5-DCP	163.00	No	0.2	3.91	23.62	96.05
Benzophenone-3	BP-3	228.26	No	0.4	100.08	424.10	100
Bisphenol A	BPA	228.29	No	0.4	1.36	5.97	86.44
Butyl paraben	BuPB	194.23	Yes	0.2	1.88	10.03	77.97
Methyl paraben	MePB	152.15	Yes	1.0	187.95	1227.27	100
Propyl paraben	PrPB	180.20	Yes	0.2	44.66	246.58	98.31
Triclosan	TCS	289.54	No	2.3	16.34	52.68	77.97
Abbreviations: GM, geometric mean; LOD, limit of detection. Concentrations < LOD were assigned a value equal to one-half the LOD.							

**Figure 1:**
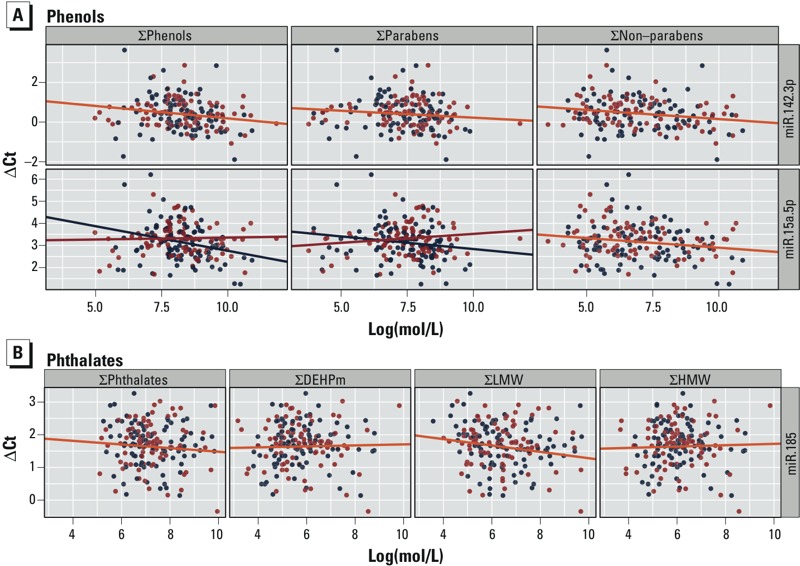
Significant associations between miRNA expression and EDC burden for (*A*) phenols and (*B*) phthalates. Blue = female; red = male; orange = overall. Association was plotted for males and females separately if there was a significant interaction between EDC burden and infant sex on miRNA levels. Estimated change in ∆Ct for a 1-unit increase in log(mol/L) EDC burden adjusting for maternal age, maternal ethnicity, and self-reported maternal smoking (yes/no), and infant sex, as well as 95% CI (see Supplemental Material, Tables S3 and S4). *p < 0.05 in adjusted model.

*Associations between grouped phthalate and phenol measurements and miRNA expression in placenta.* Two miRNAs were significantly associated with additive phenols, and miR-185 was associated with ΣLMW (Figure 1; see also Supplemental Material, Tables S3 and S4). A log(mol/L) increase in Σphenols was associated with a 0.13 [95% confidence interval (CI): –0.23, –0.03] decrease in miR-142 ΔCt. Expression of miR-142 seemed to be induced by the nonparabens, which were also significantly associated this miRNA (–0.09; 95% CI: –0.17, –0.02). Modeling miRNA expression as a function of each phenol independently, it appeared that the association with these estimates of phenol burden may be driven by benzophenone-3 (BP-3) exposure (–0.08; 95% CI: –0.14, –0.02; see Supplemental Material, Figure S5). Evaluation of the contribution of any individual metabolite is complicated by the concurrence of these compounds in consumer products, and different compounds may not share similar modes of action, and therefore must be interpreted with caution. Previously, we reported a significant weak inverse correlation between BP-3 and 2,5-dichlorophenol (2,5-DCP) (ρ = –0.24) and stronger positive correlation with BuPB (ρ = 0.28), which is also used in sunscreens and cosmetics (LaRocca et al. 2014). Levels of miR-15a-5p were also found to significantly decrease with a log(mol/L) increase of Σnonparabens [–0.09 (95% CI: –0.16, –0.01)]. Infant sex significantly modified the association between miR-15a-5p levels and both Σphenols and Σparabens. An increase in the urine concentrations of Σphenols was associated with a significant decrease only in miR-15a-5p in the placenta among female infants (–0.22; 95% CI: –0.38, –0.07). We additionally detected a significant association between miR-128 expression and both Σphenols and Σparabens (0.11; 95% CI: 0.02, 0.20 and 0.09; 95% CI: 0.01, 0.17, respectively). However, after the removal of two expression outliers that could not be explained by the sample characteristics, we did not detect a significant change (0.20; 95% CI: –0.04, 0.09 and 0.10; 95% CI: –0.04, 0.08, respectively). More than half of the miRNAs were associated (*p* < 0.05) with one of the measured phenols in our adjusted models (see Supplemental Material, Figure S5). Most of these miRNAs were either positively associated with BPA levels or negatively associated with BP-3.

We also modeled miRNA expression as a function of each additive phthalate group (Figure 1; see also Supplemental Material, Table S4). In contrast to phenols, only one miRNA was significantly associated with additive phthalates. A log(mol/L) increase in ΣLMW phthalates was significantly associated with a 0.10 (95% CI: –0.18, –0.01) decrease in miR-185 expression. This association may be driven by MEP (monoethyl phthalate) exposure, which demonstrated the strongest inverse association with miR-185 expression when modeled independently (–0.08; 95% CI: –0.15, –0.01; see Supplemental Material, Figure S5). However, this association may also reflect the impact of other LMW compounds, MiBP (mono-isobutyl phthalate) and MnBP (mono-*n*-butyl phthalate), which have a significant moderate positive correlation with MEP (LaRocca et al. 2014). Infant sex did not modify the association between any miRNA and phthalate summation. Ten of the miRNAs were associated (*p* < 0.05) with at least one of the individual phthalates (see Supplemental Material, Figure S5). A majority of these miRNAs were associated with at least MCOP (monocarboxyisooctyl phthalate).

Finally, we investigated a possible additive interaction between phthalates and phenols together on placenta miRNA expression. The interaction between Σphenols and Σphthalates on expression was not significant for any of the miRNAs (data not shown).

**Figure 2:**
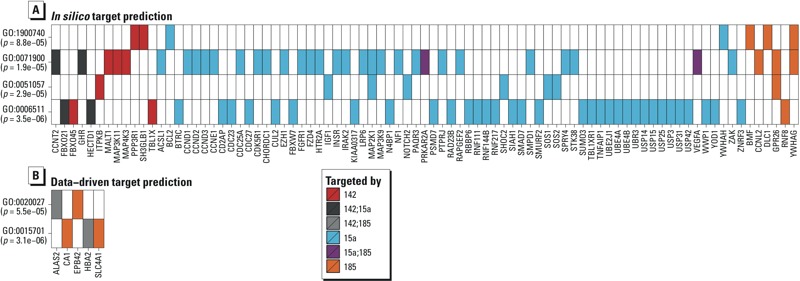
Gene ontology (GO) enrichment. Genes contributing to the enrichment (unadjusted *p* < 0.0001) of biological processes among predicted targets of miRNAs associated with EDC burden (miR-185, miR-142-3p, miR-15a-5p). Color corresponds to the miRNA(s) targeting the gene.

*Predicted mRNA targets and potentially affected pathways. In silico* target prediction software was used among mRNA (messenger RNA) targets of the three miRNAs that were significantly associated with phenol or phthalate levels (miR-185, miR-142-3p, miR-15a-5p). We investigated biological process enrichment among the genes that had at least four databases linking them to one of the three miRNAs. Table 4 lists the 19 biological pathways enriched among these putative mRNA targets. The genes with expression significantly correlated with at least one of the miRNA associated with EDC burden showed near significant (*q* = 0.07) enrichment for bicarbonate transport after adjusting for multiple testing (Table 5, Figure 2). For each miRNA, we additionally identified potential targets based on significant Spearman correlations between miRNA levels and expression across the genome (25,642 RefSeq genes). After adjusting for multiple testing, 10 genes were found to be significantly correlated with miR-142-3p, 20 were correlated with miR-185, and miR-15a-5p was not associated with any genes (see Supplemental Material, Figure S6). In all cases, miRNA levels were inversely correlated with gene expression, with Spearman correlations ranging between –0.58 and –0.46. None of these genes overlapped with the putative targets predicted *in silico*.

**Table 4:** Gene ontology (GO) biological processes among genes predicted in silico to be targeted by miRNAs associated with EDC burden (miR-185, miR-142-3p, miR-15a-5p).

GO term	Annotated	Significant	Expected	*p*‑Value	FDR *q*‑value
Ubiquitin-dependent protein catabolic process	403	39	17.43	3.50E-06	0.08
Regulation of protein serine/threonine kinase activity	366	35	15.83	1.90E-05	0.22
Positive regulation of small GTPase mediated signal transduction	30	8	1.3	2.90E-05	0.22
Positive regulation of protein insertion into mitochondrial membrane involved in apoptotic signaling pathway	26	7	1.12	8.80E-05	0.51
Heterophilic cell–cell adhesion	29	7	1.25	1.90E-04	0.88
Insulin-like growth factor receptor signaling pathway	32	8	1.38	2.60E-04	0.93
Axonal fasciculation	15	5	0.65	3.10E-04	0.93
Negative regulation of cellular macromolecule biosynthetic process	948	64	41	3.20E-04	0.93
Cellular response to insulin stimulus	207	20	8.95	5.40E-04	1.00
Regulation of sodium ion transmembrane transport	25	6	1.08	5.60E-04	1.00
Regulation of translational elongation	10	4	0.43	5.90E-04	1.00
Protein dephosphorylation	140	16	6.05	6.20E-04	1.00
Metencephalon development	84	13	3.63	6.20E-04	1.00
Embryonic epithelial tube formation	106	13	4.58	6.50E-04	1.00
Positive regulation of mesenchymal cell proliferation	36	7	1.56	7.60E-04	1.00
Platelet-derived growth factor receptor signaling pathway	36	7	1.56	7.60E-04	1.00
Smooth muscle tissue development	18	5	0.78	8.00E-04	1.00
Negative regulation of cell proliferation	529	39	22.88	9.80E-04	1.00
Neuron projection guidance	347	30	15.01	9.90E-04	1.00
FDR, false discovery rate.					

**Table 5:** Gene ontology (GO) biological processes among genes significantly correlated with miRNA associated with EDC burden (miR-185, miR-142-3p, miR-15a-5p).

GO term	Annotated	Significant	Expected	*p*‑Value	FDR *q*‑Value
Bicarbonate transport	26	3	0.03	3.10E-06	0.07
Hemoglobin metabolic process	10	2	0.01	5.50E-05	0.64
Iron ion homeostasis	84	3	0.1	1.10E-04	0.85
Small molecule metabolic process	2,610	7	2.99	1.84E-03	1.00
FDR, false discovery rate.					

*Associations with birth outcomes.* Although individual EDC concentrations were not associated with birth outcomes in this cohort (LaRocca et al. 2014), we wanted to evaluate whether the three miRNAs that were significantly associated with EDC levels were in turn associated with birth outcomes. However, we did not observe any associations between miRNA expression and gestational age, birth weight, or birth length in our adjusted models (see Supplemental Material, Table S5).

## Discussion

To our knowledge, this study is the first of its kind to identify the associations between prenatal phthalate and phenol exposure and miRNA expression in placenta. Recently, there has been an increased focus on the study of the suspected EDCs phthalates and phenols because of their high production volumes and wide-spread use in consumer products. Our results add to the body of evidence that epigenetic mechanisms are important modes of action in response to adverse gestational environments, specifically with EDC exposure.

The rationale for analyzing miRNA expression in placenta tissue was based on several factors. It has previously been suggested that several EDCs have the ability to cross the placental barrier (Takahashi and Oishi 2000). The placenta plays a critical role in fetal growth by performing many critical physiological functions, including mediating the exchange of respiratory gases, water, and nutrients, and acting as an endocrine organ to produce a number of hormones, cytokines, and signaling molecules (Jansson and Powell 2007). It is believed to be important in the development of pregnancy complications, given its role in regulating exchange between maternal and fetal blood and nutrients. Importantly, one of the few prior studies to investigate the effects of BPA on miRNA expression was performed in placenta cell lines (Avissar-Whiting et al. 2010).

We found several individual phenols and phthalates to be associated with miRNA expression in placenta. However, considering that women are concurrently exposed to several phthalates and phenols simultaneously, we used a continuous summative measurement of specific EDC categories of compounds with demonstrated shared variation, such as HWM phthalates. Although three miRNAs were significantly associated with phthalates or phenols, no significant interactions among phthalates and phenols on miRNA was observed. Our data do not indicate any synergistic or antagonistic impact of phthalate and phenol burden on miRNA levels.

We found three miRNAs for which we detected a significant association with either phenol or phthalate levels on expression: miR-142-3p, miR15a-5p, and miR-185. We input these three miRNAs into our *in silico* analyses to investigate potential targets and affected pathways. Gene enrichment analysis revealed several biological processes associated with the potential mRNA targets of these three miRNAs. These processes included regulation of protein serine/threonine kinase activity and positive regulation of protein insertion into mitochondrial membrane involved in apoptotic signaling pathway. Serine/threonine kinase activity is a major component of the apoptotic pathway. Importantly, prior research has demonstrated that exposure to physiologically relevant levels of BPA induces apoptosis in human cytotrophoblasts (Benachour and Aris 2009). Additionally, phthalate exposure has been implicated in oxidative stress in placental cells (Tetz et al. 2013), and MEHP is a well-known Sertoli cell toxicant that disrupts germ cell apoptosis following early-life exposure in rats (Richburg and Boekelheide 1996).

Other biological processes that were associated with the overall list of potential mRNA targets included cellular response to insulin stimulus and insulin-like growth factor receptor signaling pathway. Importantly, gestational diabetes has been implicated in abnormal insulin receptor signaling in placenta (Colomiere et al. 2009). Other targeted processes include the regulation of metencephalon development, and embryonic epithelial tube formation. Overall, our data indicate that prenatal phthalate and phenol exposure may interfere with several biological processes that have been previously implicated in placental and fetal health.

Additionally, differential expression of miR-185 has been observed in placenta samples from preeclamptic pregnancies compared with non-preeclamptic pregnancies (Ishibashi et al. 2012; Wang W et al. 2012). Prior research indicates that oxidative stress in placentas from preeclamptic pregnancies leads to compromised calcium homeostatis, and preeclampsia has also been associated with disrupted iron ion homeostasis (Haché et al. 2011; Rayman et al. 2002). Our data indicate that the biological processes among genes significantly correlated with miRNA associated with phthalate and phenol burden include iron ion homeostasis and small molecule metabolic process. The placenta is the site of exchange between the mother and fetus, and the placenta transports calcium ions actively, and is a site of metabolism for small amino acids (Pitkin 1985). Disruption of these pathways may negatively affect the health of the placenta and fetus. miR-185 may represent a critical target in mediating these processes as a result of an adverse gestational environment, because its expression is increased in preeclamptic placentas compared with healthy pregnancies, and is increased in placentas from our study with higher phthalate and phenol exposure levels compared with lower exposure levels. Several other miRNAs that were analyzed in this study have been associated with other exposures as well. For instance, maternal smoking has been associated with downregulation of mir-16 in human placenta (Maccani et al. 2010). Exposure to particulate matter has been associated with altered expression of miR-128 (Bollati et al. 2015). Additionally, exposure to perfluorooctanoic acid is associated with circulating miR-26b levels in fluorochemical plant workers (Wang J et al. 2012).

A limitation of this study is the use of a single urine measurement. Several prior studies have reported estimates of reproducibility of select phthalate and phenol biomarker concentrations in pregnant women and women of reproductive age (Adibi et al. 2008; Braun et al. 2012; Peck et al. 2010). Although paraben levels exhibit some variability, a single urine measurement during pregnancy may suitably represent gestational exposure (Smith et al. 2012). Reproducibility in phthalate biomarker measurements also varies depending on the study and chemical (Adibi et al. 2008; Irvin et al. 2010). Other environmental or hormonal exposures not analyzed in this study may also confound miRNA expression. Cellular heterogeneity also represents another limitation of the study. Differences in cell populations can affect methylation in blood, and there are currently reference-free methods to adjust for cellular heterogeneity (Houseman et al. 2012, 2014). However, to date there are no methods to adjust for cell populations in the placenta, and disparities in methylation due to alterations in cell populations may reflect an outcome of EDC exposure, rather than a confounder. Although miRNA alterations following exposure to exogenous toxicants may not in turn cause the full expected transcriptional responses (Rager et al. 2014), we propose that the predicted pathway-level changes may still represent critical targets of EDC disruption in the placenta. miRNAs can target several mRNAs, and may affect protein level at the translational level rather than mRNA degradation. It is possible that some of the target predictions would be false positives in this respect. The genes that were significantly associated with miRNAs were not represented in the *in silico* analysis, which may reflect their repression via indirect routes or may indicate noncausal associations. Our study also exhibits several considerable strengths, the first of which was the measurement of EDC exposure during the first trimester, which represents a critical window of exposure for implications in adverse health outcomes later in life (Symonds et al. 2007). This is also the first study of its kind to analyze associations between several miRNAs and prenatal phthalate and phenol exposure in humans. Notably, one study reported that placental miRNA is associated with early neurobehavioral outcomes (Maccani et al. 2013). Given that early-life perturbations can influence disease development later in life, we propose that future research is needed to assess developmental outcomes during childhood and adulthood in this cohort.

## Conclusions

Our data suggests that prenatal EDC exposure is associated with altered miRNA expression in the placenta. Given the ability for miRNAs to target important cellular pathways, the regulation of miRNAs is very important for placental and fetal growth. Differential expression of miRNAs associated with EDC exposure may be implicated in disrupted biological processes. Overall, we propose that miRNA regulation is a potentially significant epigenetic toxicity mechanism of prenatal phenol and phthalate exposure, which warrants future investigations to confirm.

## Supplemental Material

(1.6 MB) PDF
