# Supplementary material for: First-Trimester Urine Concentrations of Phthalate Metabolites and Phenols and Placenta miRNA Expression in a Cohort of U.S. Women
Source: Environ Health Perspect. 2015 Jun 19;124(3):380–7. doi: 10.1289/ehp.1408409 (PMC4786977; doi:10.1289/ehp.1408409)
Supplement: (1.6 MB) PDF [file ehp.1408409.s001.acco.pdf]

Environ Health Perspect
DOI: 10.1289/ehp.1408409

Note to Readers: *EHP* strives to ensure that all journal content is accessible to all readers. However, some figures and Supplemental Material published in *EHP* articles may not conform to 508 standards due to the complexity of the information being presented. If you need assistance accessing journal content, please contact ehp508@niehs.nih.gov. Our staff will work with you to assess and meet your accessibility needs within 3 working days.

Supplemental Material

First-Trimester Urine Concentrations of Phthalate Metabolites and Phenols and Placenta miRNA Expression in a Cohort of U.S. Women

Jessica LaRocca, Alexandra M. Binder, Thomas F. McElrath, and Karin B. Michels

Table of Contents

Figure S1. Study population for women eligible for analysis of first-trimester urinary phthalate and phenol concentrations with miRNA alterations in the placenta. Urinary phthalate and phenol concentrations were analyzed in 196 women who were enrolled in both the Predictors of Preeclampsia Study (POPS) and Epigenetic Birth Cohort (EBC) and had a first-trimester urine sample. Women without available placenta for RNA extraction were excluded from relevant analyses (N=17), giving a total population of N=179. The participants for the original pilot project were nested in this population (N=48).

Table S1. Estimates and p values for pilot project results comparing miRNA expression with grouped phthalate and phenol categories.

Table S2. List of analyzed miRNAs and accession numbers

Figure S2. Coefficient of variation (CV) for a subset of samples run in triplicate for all miRNAs analyzed.

Figure S3. Distribution of phenols and phthalates among the samples chosen for the pilot study and the full sample.

Figure S4. Pearson correlation (r) between the miRNA levels estimated from the pilot and the full study. *p<0.001.

Table S3. Associations between Placental miRNA Expression and Grouped Maternal Phenol Urine Concentrations. Estimated change in ΔCt for a one unit increase in log(mol/L) EDC burden in multivariable models.

Table S4. Associations between Placental miRNA Expression and Additive Maternal Phthalate Urine Concentrations. Estimated change in ΔCt expression for a one unit increase in log(mol/L) EDC burden in multivariable models.

Figure S5. Association between miRNA Expression and each EDC Metabolite Independently. Each cell corresponds to a test for the association between an individual metabolite (columns) and the expression of a specific miRNA (rows). The cell color corresponds to the change in ΔCt miRNA expression with a one log(mol/L) increase in metabolite adjusting for maternal age, maternal ethnicity, and self-reported maternal smoking (Yes/No), and infant-sex; dots indicate $p<0.05$ for the association between metabolite and EDC in the multivariable model; green=non-paraben phenol, orange=paraben; pink=LMW phthalate; turquoise=HMW non-DEHP phthalate; purple=HMW DEHP phthalate.

Figure S6. MicroRNA and gene expression levels among the subset of significant pairwise Spearman correlations identified among a subset of samples run on Affymetrix GeneChip® Human Gene 2.0 ST Array after adjusting for multiple testing.

Table S5. Correlations between birth outcomes and miRNAs significant among EDC profiles.

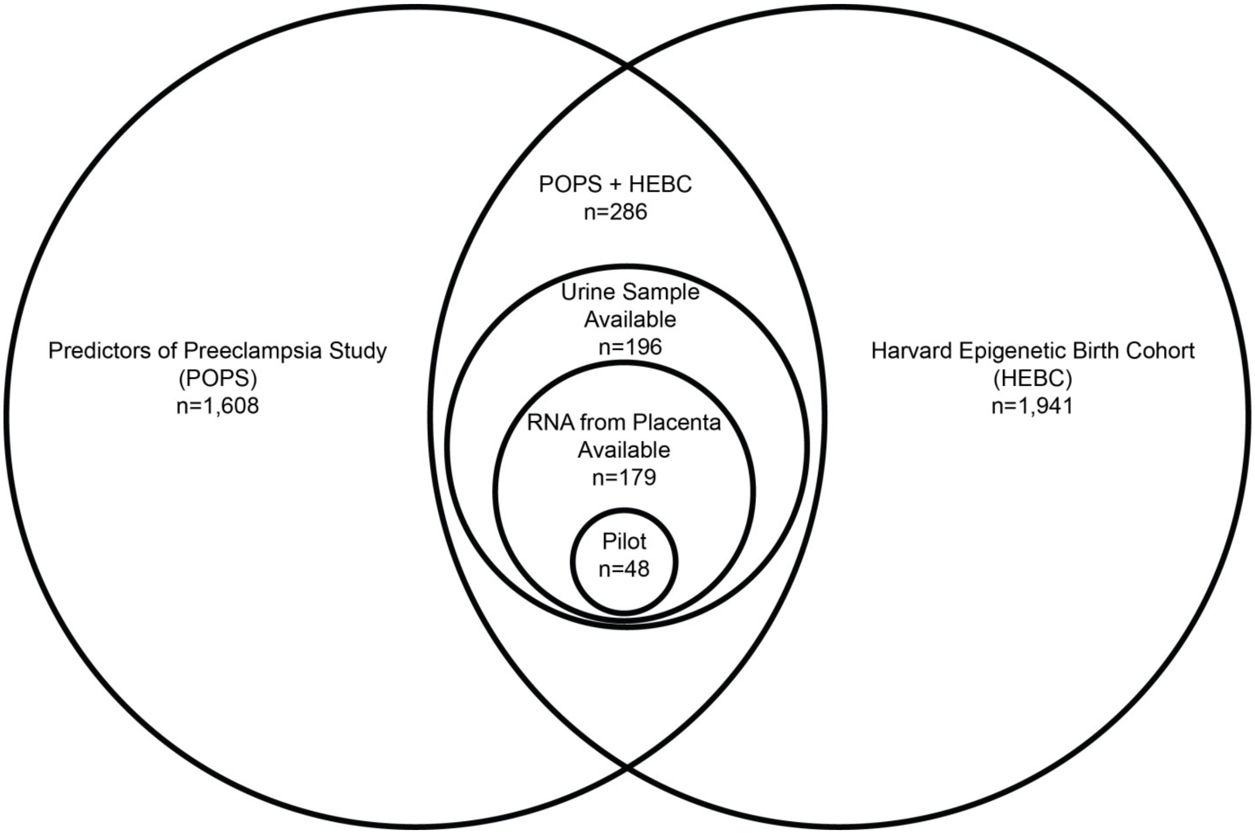

Figure S1. Study population for women eligible for analysis of first-trimester urinary phthalate and phenol concentrations with miRNA alterations in the placenta. Urinary phthalate and phenol concentrations were analyzed in 196 women who were enrolled in both the Predictors of Preeclampsia Study (POPS) and Epigenetic Birth Cohort (EBC) and had a first-trimester urine sample. Women without available placenta for RNA extraction were excluded from relevant analyses (N=17), giving a total population of N=179. The participants for the original pilot project were nested in this population (N=48).

Table S1. Estimates and p values for pilot project results comparing miRNA expression with grouped phthalate and phenol categories.

miRNA	ΣPhenols pval	ΣPhenols estimate	Parabens pval	Parabens estimate	Non Parabens pval	ΣParabens estimate	ΣPhthalates pval	ΣPhthalates estimate	DEHP metabolites pval	DEHP Metabolites estimate	LMW pval	LMW estimate	HMW pval	HMW estimate
hsa.miR.19b.3p	0.00	-0.44	0.02	-0.35	0.01	-0.37	0.15	0.22	0.08	0.26	0.35	0.14	0.11	0.24
hsa.miR.19a.3p	0.01	-0.40	0.03	-0.33	0.03	-0.32	0.06	0.28	0.03	0.33	0.22	0.19	0.03	0.32
hsa.miR.106b.5p	0.01	-0.39	0.04	-0.30	0.05	-0.29	0.54	0.09	0.26	0.17	0.88	0.02	0.28	0.16
hsa.miR.142.3p	0.01	-0.38	0.05	-0.30	0.03	-0.32	0.06	0.28	0.02	0.36	0.17	0.21	0.02	0.35
hsa.miR.17.5p	0.01	-0.38	0.03	-0.32	0.08	-0.26	0.32	0.15	0.10	0.25	0.85	0.03	0.14	0.22
hsa.miR.16.5p	0.02	-0.36	0.02	-0.34	0.14	-0.22	0.08	0.27	0.01	0.39	0.44	0.12	0.01	0.38
hsa.miR.144.3p	0.02	-0.35	0.17	-0.21	0.05	-0.29	0.07	0.27	0.02	0.35	0.19	0.20	0.02	0.36
hsa.miR.18a.5p	0.03	-0.33	0.08	-0.26	0.09	-0.26	0.05	0.30	0.01	0.38	0.14	0.22	0.01	0.39
hsa.miR.101.3p	0.03	-0.33	0.11	-0.24	0.03	-0.32	0.06	0.28	0.12	0.24	0.10	0.25	0.13	0.23
hsa.miR.93.5p	0.03	-0.33	0.18	-0.20	0.06	-0.28	0.17	0.21	0.08	0.26	0.59	0.08	0.10	0.25
hsa.miR.15a.5p	0.03	-0.33	0.04	-0.31	0.35	-0.14	0.08	0.26	0.06	0.28	0.14	0.22	0.05	0.29
hsa.miR.103a.3p	0.03	-0.32	0.04	-0.31	0.11	-0.24	0.25	0.17	0.28	0.17	0.43	0.12	0.25	0.17
hsa.miR.141.3p	0.04	-0.31	0.13	-0.23	0.00	-0.42	0.62	0.08	0.99	0.00	0.38	0.13	0.94	0.01
hsa.miR.195.5p	0.04	-0.31	0.09	-0.26	0.13	-0.23	0.14	0.22	0.03	0.33	0.48	0.11	0.03	0.32
hsa.miR.92a.3p	0.05	-0.30	0.05	-0.29	0.43	-0.12	0.13	0.23	0.06	0.29	0.65	0.07	0.06	0.28
hsa.miR.186.5p	0.06	-0.28	0.20	-0.19	0.01	-0.39	0.10	0.25	0.25	0.17	0.19	0.20	0.23	0.18
hsa.miR.130a.3p	0.07	-0.28	0.17	-0.21	0.22	-0.18	0.65	0.07	0.70	0.06	0.90	0.02	0.77	0.05
hsa.miR.30b.5p	0.07	-0.27	0.08	-0.26	0.04	-0.31	0.14	0.23	0.23	0.18	0.38	0.13	0.24	0.18
hsa.miR.20a.5p	0.07	-0.27	0.21	-0.19	0.14	-0.22	0.68	0.06	0.32	0.15	0.93	0.01	0.35	0.14
hsa.miR.140.3p	0.08	-0.27	0.10	-0.25	0.20	-0.19	0.39	0.13	0.46	0.11	0.70	0.06	0.59	0.08
hsa.miR.15b.5p	0.10	-0.25	0.14	-0.22	0.58	-0.08	0.05	0.29	0.01	0.36	0.61	0.08	0.01	0.36
hsa.miR.425.5p	0.11	-0.24	0.16	-0.21	0.04	-0.30	0.14	0.23	0.12	0.23	0.15	0.22	0.12	0.23
hsa.let.7f.5p	0.11	-0.24	0.20	-0.19	0.28	-0.16	0.79	0.04	0.48	0.11	0.36	-0.14	0.66	0.07
hsa.miR.374a.5p	0.11	-0.24	0.18	-0.20	0.11	-0.24	0.69	0.06	0.92	0.01	0.79	-0.04	0.87	0.02
hsa.miR.32.5p	0.12	-0.23	0.25	-0.17	0.20	-0.20	0.47	0.11	0.75	0.05	0.31	0.15	0.69	0.06
hsa.miR.185.5p	0.13	-0.23	0.19	-0.20	0.63	-0.07	0.15	0.22	0.07	0.27	0.69	0.06	0.06	0.28
hsa.miR.30a.5p	0.14	-0.22	0.09	-0.25	0.29	-0.16	0.51	0.10	0.67	0.06	0.95	-0.01	0.64	0.07
hsa.miR.128	0.15	-0.22	0.27	-0.17	0.60	-0.08	0.19	0.20	0.21	0.19	0.96	-0.01	0.17	0.21
hsa.miR.223.3p	0.15	-0.22	0.24	-0.18	0.25	-0.17	0.39	0.13	0.39	0.13	0.98	0.00	0.38	0.13
hsa.miR.424.5p	0.15	-0.22	0.13	-0.23	0.31	-0.15	0.25	0.18	0.30	0.16	0.80	0.04	0.27	0.17
hsa.miR.150.5p	0.16	-0.21	0.07	-0.27	0.51	-0.10	0.53	0.10	0.29	0.16	0.72	-0.05	0.37	0.14
hsa.miR.143.3p	0.16	-0.21	0.19	-0.20	0.76	-0.05	0.03	0.32	0.07	0.28	0.12	0.24	0.10	0.25
hsa.miR.142.5p	0.17	-0.21	0.27	-0.17	0.32	-0.15	0.59	0.08	0.59	0.08	0.31	0.15	0.58	0.08
hsa.miR.25.3p	0.18	-0.20	0.40	-0.13	0.51	-0.10	0.11	0.24	0.04	0.31	0.61	0.08	0.04	0.30
hsa.miR.22.3p	0.18	-0.20	0.61	-0.08	0.02	-0.34	0.02	0.36	0.10	0.25	0.01	0.39	0.07	0.27
hsa.miR.28.5p	0.21	-0.19	0.27	-0.17	0.37	-0.14	0.06	0.29	0.37	0.14	0.23	0.18	0.43	0.12

miRNA	ΣPhenols pval	ΣPhenols estimate	Parabens pval	Parabens estimate	Non Parabens pval	ΣParabens estimate	ΣPhthalates pval	ΣPhthalates estimate	DEHP metabolites pval	DEHP Metabolites estimate	LMW pval	LMW estimate	HMW pval	HMW estimate
hsa.miR.194.5p	0.22	-0.19	0.21	-0.19	0.27	-0.17	0.63	0.07	0.46	0.11	0.92	0.02	0.44	0.12
hsa.let.7g.5p	0.22	-0.19	0.11	-0.24	0.86	-0.03	0.20	0.19	0.29	0.16	0.72	0.05	0.30	0.16
hsa.miR.30c.5p	0.23	-0.18	0.09	-0.26	0.60	-0.08	0.44	0.12	0.65	0.07	0.92	-0.02	0.53	0.10
hsa.miR.210	0.24	-0.18	0.30	-0.16	0.56	-0.09	0.58	-0.08	0.60	-0.08	0.87	-0.03	0.60	-0.08
hsa.miR.24.3p	0.26	-0.17	0.16	-0.21	0.36	-0.14	0.34	0.15	0.54	0.09	0.82	0.04	0.53	0.10
hsa.miR.29b.3p	0.27	-0.17	0.47	-0.11	0.19	-0.20	0.93	0.01	0.84	-0.03	0.80	0.04	0.90	-0.02
hsa.miR.30e.5p	0.28	-0.16	0.13	-0.23	0.39	-0.13	0.76	0.05	0.87	0.02	0.74	-0.05	0.86	0.03
hsa.miR.26b.5p	0.30	-0.16	0.18	-0.20	0.54	-0.09	0.40	0.13	0.61	0.08	0.73	-0.05	0.61	0.08
hsa.miR.191.5p	0.34	-0.15	0.19	-0.20	0.60	-0.08	0.87	0.03	0.97	-0.01	0.49	-0.10	0.95	0.01
hsa.let.7d.5p	0.34	-0.14	0.13	-0.23	0.62	-0.08	0.44	0.12	0.40	0.13	0.97	-0.01	0.54	0.09
hsa.miR.302c.3p	0.35	-0.14	0.73	-0.05	0.57	-0.09	0.02	0.34	0.01	0.40	0.24	0.18	0.01	0.41
hsa.miR.7.5p	0.37	-0.14	0.98	0.00	0.11	-0.24	0.51	0.10	0.47	0.11	0.83	-0.03	0.50	0.10
hsa.miR.126.3p	0.39	-0.13	0.09	-0.25	0.71	-0.06	0.73	-0.05	0.91	0.02	0.17	-0.21	0.83	0.03
hsa.miR.302a.3p	0.41	-0.13	0.43	-0.12	0.49	-0.10	0.26	-0.17	0.65	-0.07	0.17	-0.21	0.60	-0.08
hsa.miR.26a.5p	0.43	-0.12	0.14	-0.22	0.97	0.00	0.68	0.06	0.91	-0.02	0.54	-0.09	0.99	0.00
hsa.miR.302b.3p	0.43	-0.12	0.33	-0.15	0.79	-0.04	0.04	0.31	0.15	0.22	0.08	0.26	0.15	0.22
hsa.miR.27a.3p	0.46	-0.11	0.77	-0.04	0.13	-0.23	0.27	0.17	0.54	0.09	0.53	0.10	0.58	0.08
hsa.miR.27b.3p	0.47	-0.11	0.19	-0.20	0.74	-0.05	0.27	0.17	0.56	0.09	0.68	0.06	0.62	0.08
hsa.let.7a.5p	0.47	-0.11	0.18	-0.20	0.97	-0.01	0.42	0.12	0.54	0.09	0.78	-0.04	0.56	0.09
hsa.miR.9.5p	0.49	-0.10	0.87	0.02	0.17	-0.21	0.25	0.17	0.60	0.08	0.48	0.11	0.63	0.07
hsa.miR.125a.5p	0.51	-0.10	0.44	-0.12	0.44	-0.12	0.33	0.15	0.60	0.08	0.89	0.02	0.60	0.08
hsa.miR.30d.5p	0.52	-0.10	0.38	-0.13	0.61	-0.08	0.64	0.07	0.91	-0.02	0.96	0.01	0.92	-0.02
hsa.miR.125b.5p	0.52	-0.10	0.40	-0.13	0.66	-0.07	0.88	0.02	1.00	0.00	0.66	-0.07	1.00	0.00
hsa.miR.200c.3p	0.53	-0.10	0.36	-0.14	0.38	-0.13	0.89	0.02	0.60	-0.08	0.91	-0.02	0.56	-0.09
hsa.miR.181b.5p	0.54	-0.09	0.12	-0.23	0.77	-0.04	0.95	0.01	0.70	-0.06	0.73	-0.05	0.78	-0.04
hsa.miR.124.3p	0.57	0.09	0.60	0.08	0.33	0.15	0.77	0.05	0.85	0.03	0.53	0.10	0.91	0.02
hsa.let.7b.5p	0.57	0.09	0.85	-0.03	0.57	0.09	0.53	-0.10	0.33	-0.15	0.23	-0.18	0.21	-0.19
hsa.miR.122.5p	0.57	-0.09	0.26	-0.17	0.83	0.03	0.43	-0.12	0.95	-0.01	0.04	-0.30	0.80	-0.04
cel.miR.39.3p	0.60	0.08	0.60	-0.08	0.50	0.10	0.19	0.20	0.22	0.19	0.42	0.12	0.19	0.20
hsa.miR.151a.5p	0.64	-0.07	0.88	-0.02	0.35	-0.14	0.44	0.12	0.94	0.01	0.86	0.03	0.91	0.02
hsa.miR.99a.5p	0.65	-0.07	0.31	-0.15	0.83	0.03	0.12	0.23	0.14	0.22	0.22	0.18	0.22	0.19
hsa.miR.96.5p	0.65	-0.07	0.59	-0.08	0.94	-0.01	0.76	-0.05	0.37	-0.14	0.64	0.07	0.57	-0.09
hsa.let.7i.5p	0.68	-0.06	0.57	-0.09	0.98	0.00	0.93	0.01	0.84	0.03	0.46	-0.11	0.93	0.01
hsa.miR.423.5p	0.69	0.06	0.65	0.07	0.78	-0.04	0.96	0.01	0.53	-0.10	0.93	-0.01	0.55	-0.09
hsa.let.7e.5p	0.69	-0.06	0.49	-0.11	0.88	-0.02	0.66	0.07	0.73	0.05	0.48	-0.11	0.79	0.04
hsa.let.7c	0.70	-0.06	0.39	-0.13	0.89	0.02	0.67	0.07	0.53	0.10	0.41	-0.13	0.73	0.05
hsa.miR.222.3p	0.78	-0.04	0.33	-0.15	0.29	0.16	0.26	0.17	0.50	0.10	0.78	0.04	0.55	0.09
hsa.miR.320a	0.78	0.04	0.58	-0.09	0.35	0.14	0.69	0.06	0.85	0.03	0.86	-0.03	0.85	0.03
cel.miR.39.3p.1	0.82	0.04	0.76	0.05	0.89	0.02	0.03	0.33	0.06	0.28	0.21	0.19	0.11	0.24

miRNA	ΣPhenols pval	ΣPhenols estimate	Parabens pval	Parabens estimate	Non Parabens pval	ΣParabens estimate	ΣPhthalates pval	ΣPhthalates estimate	DEHP metabolites pval	DEHP Metabolites estimate	LMW pval	LMW estimate	HMW pval	HMW estimate
hsa.miR.100.5p	0.82	-0.03	0.84	0.03	0.40	-0.13	0.42	-0.12	0.34	-0.14	0.31	-0.16	0.35	-0.14
hsa.miR.23a.3p	0.82	-0.03	0.67	-0.07	0.68	-0.06	0.21	0.19	0.73	0.05	0.58	0.09	0.63	0.07
hsa.miR.181a.5p	0.82	0.03	0.68	-0.06	0.91	-0.02	0.83	0.03	0.54	-0.09	0.27	0.17	0.65	-0.07
hsa.miR.376c.3p	0.84	-0.03	0.54	-0.09	0.42	0.12	0.18	0.21	0.20	0.19	0.42	0.12	0.25	0.18
hsa.miR.155.5p	0.87	0.02	0.75	0.05	0.86	-0.03	0.08	0.26	0.16	0.21	0.36	0.14	0.17	0.21
hsa.miR.29c.3p	0.90	-0.02	0.99	0.00	0.41	-0.13	0.82	0.04	0.94	-0.01	0.95	-0.01	0.93	-0.01
hsa.miR.196b.5p	0.93	-0.01	0.54	-0.09	0.56	0.09	0.33	0.15	0.61	0.08	0.92	0.02	0.62	0.08
hsa.miR.29a.3p	0.93	-0.01	0.90	-0.02	0.51	-0.10	0.99	0.00	1.00	0.00	0.82	-0.03	0.96	-0.01
hsa.miR.21.5p	0.95	-0.01	0.57	-0.09	0.74	0.05	0.15	0.22	0.39	0.13	0.67	0.07	0.36	0.14
hsa.miR.146a.5p	0.99	0.00	0.52	-0.10	0.71	0.06	0.77	0.05	0.59	-0.08	0.80	-0.04	0.61	-0.08
hsa.miR.23b.3p	1.00	0.00	0.61	-0.08	0.58	0.09	0.14	0.23	0.43	0.12	0.54	0.09	0.43	0.12

Pval=p value; HMW= high molecular weight phthalates; LMW= low molecular weight phthalates

Table S2. List of analyzed miRNAs and accession numbers

miRNA	Accession #
has-let-7d-5p	MIMAT0000065
has-let-7g-5p	MIMAT0000414
hsa-miR-106b-5p	MIMAT0000680
hsa-miR-125a-5p	MIMAT0000443
hsa-miR-125b-5p	MIMAT0000423
hsa-miR-126-3p	MIMAT0000445
hsa-miR-128	MIMAT0000424
hsa-miR-142-3p	MIMAT0000434
hsa-miR-143-3p	MI0000459
hsa-miR-155-5p	MIMAT0000646
hsa-miR-15a-5p	MIMAT0000068
hsa-miR-15b-5p	MIMAT0000417
hsa-miR-16-5p	MIMAT0000069
hsa-miR-17-5p	MIMAT0000070
hsa-miR-185	MIMAT0000455
hsa-miR-18a-5p	MIMAT0000072
hsa-miR-195-5p	MIMAT0000461
hsa-miR-19a-3p	MIMAT0000073
hsa-miR-200c-3p	MIMAT0000617
hsa-miR-20a-5p	MIMAT0000075
hsa-miR-21-5p	MIMAT0000076
hsa-miR-222-3p	MIMAT0000279
hsa-miR-25-3p	MIMAT0000081
hsa-miR-26b-5p	MIMAT0000083
hsa-miR-29c-3p	MIMAT0000681
hsa-miR-30a-5p	MIMAT0000087
hsa-miR-30c-5p	MIMAT0000244
hsa-miR-92a-3p	MIMAT0000092
hsa-miR-93-5p	MIMAT0000093

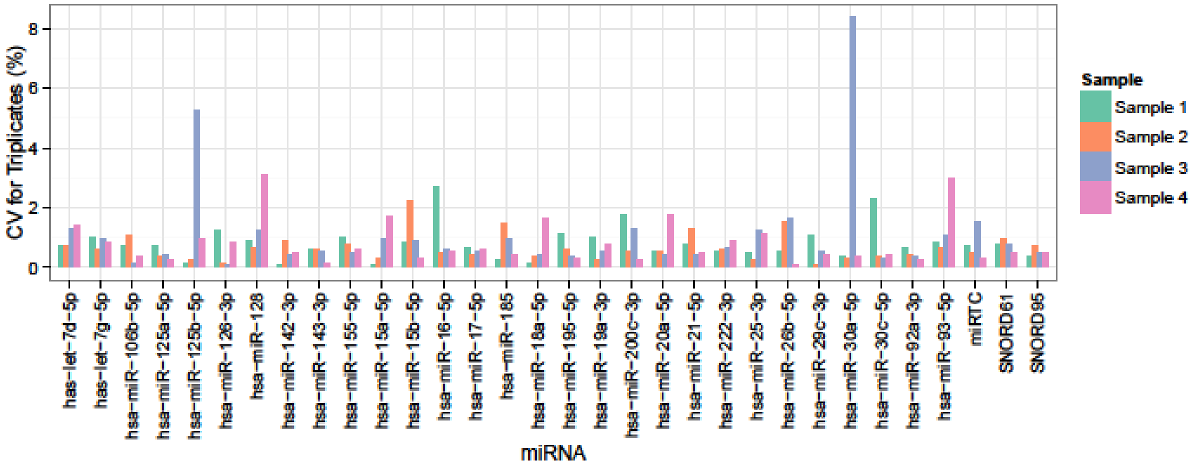

Figure S2. Coefficient of variation (CV) for a subset of samples run in triplicate for all miRNAs analyzed.

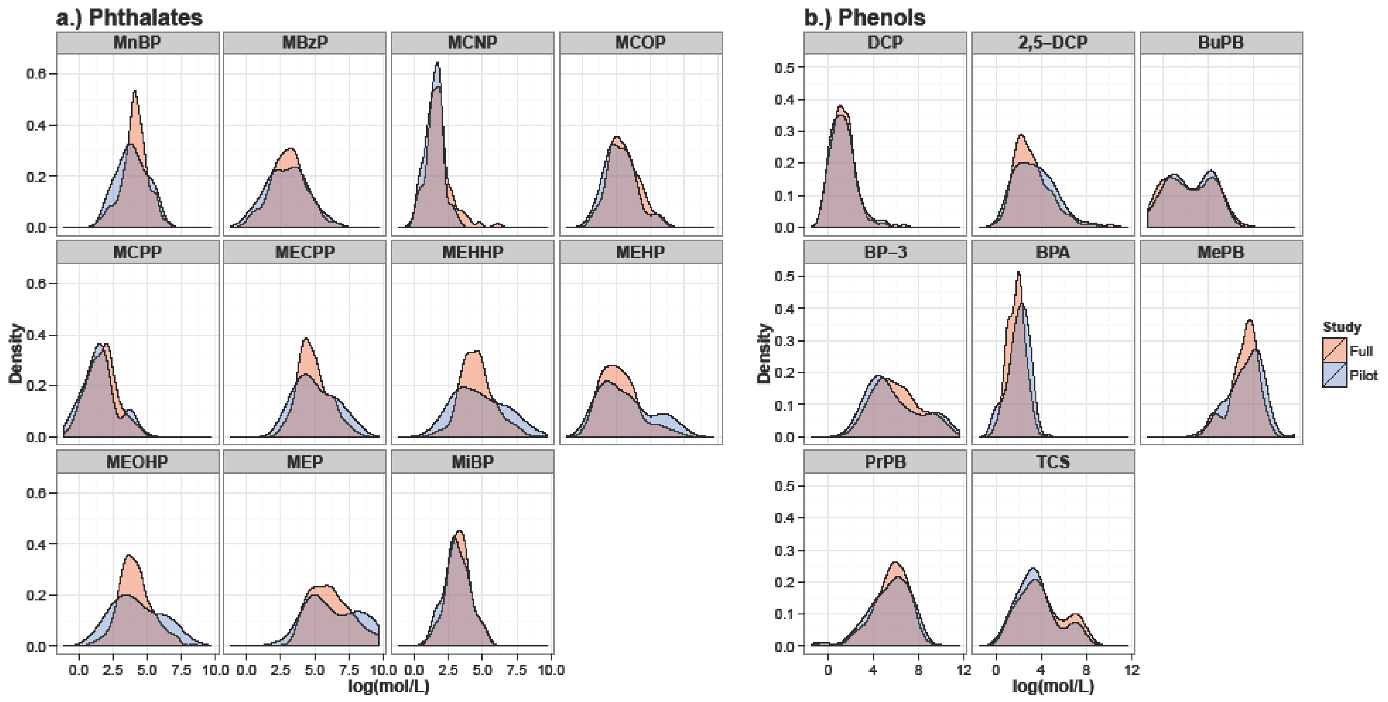

Figure S3. Distribution of phenols and phthalates among the samples chosen for the pilot study and the full sample.

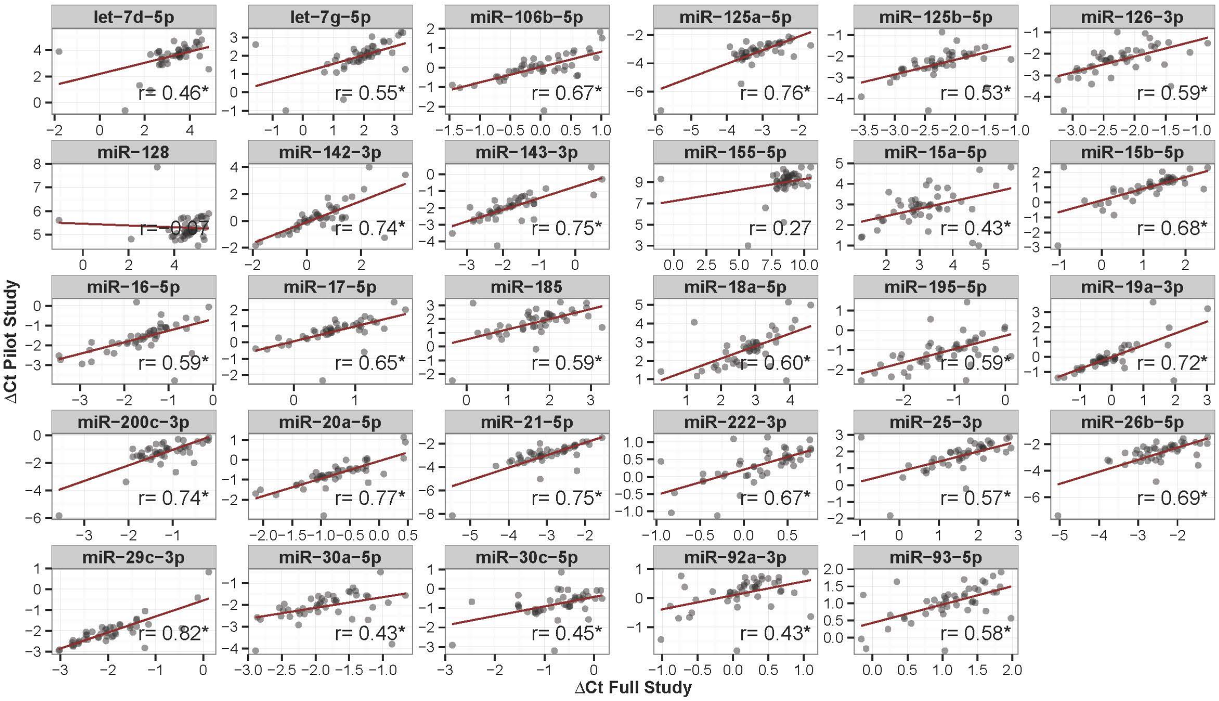

Figure S4. Pearson correlation (r) between the miRNA levels estimated from the pilot and the full study. *p<0.001.

Table S3. Associations between Placental miRNA Expression and Grouped Maternal Phenol Urine Concentrations. Estimated change in ΔCt for a one unit increase in log(mol/L) EDC burden in multivariable models.

microRNA	∑Phenols Coefficient (CI)	∑Parabens Coefficient (CI)	∑Non-Parabens Coefficient (CI)
has-let-7d-5p	0.06 (-0.05, 0.16)	0.04 (-0.06, 0.14)	0.04 (-0.04, 0.12)
has-let-7g-5p	0.03 (-0.08, 0.15)	0.00 (-0.11, 0.11)	0.02 (-0.07, 0.11)
hsa-miR-106b-5p	-0.06 (-0.12, 0.00)	-0.04 (-0.09, 0.02)	-0.04 (-0.09, 0.00)
hsa-miR-125a-5p	0.04 (-0.05, 0.13)	0.00 (-0.09, 0.08)	0.03 (-0.03, 0.10)
hsa-miR-125b-5p	0.04 (-0.04, 0.12)	0.02 (-0.05, 0.09)	0.02 (-0.04, 0.08)
hsa-miR-126-3p	0.00 (-0.07, 0.07)	-0.01 (-0.08, 0.05)	-0.01 (-0.06, 0.04)
hsa-miR-128	0.11 (0.02, 0.20)[a]	0.09 (0.01, 0.17)[a]	0.04 (-0.03, 0.10)
hsa-miR-142-3p	-0.13 (-0.23, -0.03)*	-0.07 (-0.17, 0.03)	-0.09 (-0.17, -0.02)*
hsa-miR-143-3p	-0.05 (-0.12, 0.02)	-0.01 (-0.08, 0.06)	-0.03 (-0.09, 0.02)
hsa-miR-155-5p	0.10 (-0.05, 0.25)	0.13 (-0.02, 0.27)	0.00 (-0.11, 0.11)
hsa-miR-15a-5p	M: 0.02 (–0.11, 0.15)[b]<br>F: –0.22 (–0.38, –0.07)*	M: 0.08 (–0.05, 0.21)[b]<br>F: –0.11 (–0.25, 0.02)	-0.09 (-0.16, -0.01)*
hsa-miR-15b-5p	0.03 (-0.06, 0.13)	0.00 (-0.09, 0.09)	0.03 (-0.04, 0.10)
hsa-miR-16-5p	-0.05 (-0.13, 0.03)	-0.05 (-0.12, 0.03)	-0.03 (-0.09, 0.03)
hsa-miR-17-5p	-0.04 (-0.10, 0.02)	-0.02 (-0.07, 0.04)	-0.04 (-0.08, 0.01)
hsa-miR-185	-0.01 (-0.09, 0.08)	-0.01 (-0.09, 0.07)	-0.01 (-0.07, 0.05)
hsa-miR-18a-5p	-0.03 (-0.11, 0.06)	0.00 (-0.09, 0.08)	-0.04 (-0.11, 0.02)
hsa-miR-195-5p	0.11 (-0.09, 0.32)	0.04 (-0.15, 0.24)	0.09 (-0.06, 0.25)
hsa-miR-19a-3p	-0.07 (-0.15, 0.02)	-0.02 (-0.10, 0.07)	-0.06 (-0.12, 0.01)
hsa-miR-200c-3p	0.06 (-0.02, 0.15)	0.03 (-0.05, 0.12)	0.04 (-0.03, 0.10)
hsa-miR-20a-5p	-0.03 (-0.09, 0.03)	-0.02 (-0.08, 0.04)	-0.04 (-0.08, 0.01)
hsa-miR-21-5p	0.06 (-0.05, 0.16)	0.02 (-0.08, 0.12)	0.03 (-0.04, 0.11)
hsa-miR-222-3p	0.05 (-0.01, 0.11)	0.03 (-0.03, 0.08)	0.04 (0.00, 0.08)
hsa-miR-25-3p	0.05 (-0.05, 0.14)	0.01 (-0.07, 0.10)	0.02 (-0.05, 0.09)
hsa-miR-26b-5p	0.06 (-0.04, 0.16)	0.03 (-0.07, 0.12)	0.02 (-0.05, 0.10)
hsa-miR-29c-3p	-0.02 (-0.08, 0.05)	0.01 (-0.05, 0.07)	-0.04 (-0.09, 0.01)
hsa-miR-30a-5p	0.01 (-0.06, 0.07)	0.01 (-0.06, 0.07)	-0.01 (-0.05, 0.04)
hsa-miR-30c-5p	0.04 (-0.03, 0.12)	0.04 (-0.03, 0.11)	0.01 (-0.04, 0.07)
hsa-miR-92a-3p	0.02 (-0.03, 0.08)	0.00 (-0.06, 0.05)	0.02 (-0.02, 0.06)
hsa-miR-93-5p	-0.01 (-0.08, 0.06)	0.01 (-0.06, 0.07)	-0.02 (-0.07, 0.03)

CI=Confidence Interval

[a] significant association ($p<0.05$) no longer significant after removing 2 influential points

[b] $p<0.05$ for interaction by infant sex, so association is reported separately

*$p<0.05$, adjusting for maternal age, maternal ethnicity, and self-reported maternal smoking (Yes/No), and infant-sex

Table S4. Associations between Placental miRNA Expression and Additive Maternal Phthalate Urine Concentrations. Estimated change in ΔCt expression for a one unit increase in log(mol/L) EDC burden in multivariable models.

**microRNA**	**∑Phthalates Coefficient (CI)**	**∑DEHPm Coefficient (CI)**	**∑LMW Coefficient (CI)**	**∑HMW Coefficient (CI)**
has-let-7d-5p	-0.01 (-0.14, 0.12)	-0.04 (-0.14, 0.07)	-0.01 (-0.12, 0.10)	-0.05 (-0.16, 0.07)
has-let-7g-5p	-0.04 (-0.18, 0.10)	-0.05 (-0.17, 0.07)	-0.02 (-0.14, 0.09)	-0.06 (-0.19, 0.07)
hsa-miR-106b-5p	0.00 (-0.07, 0.07)	0.05 (-0.01, 0.11)	-0.02 (-0.08, 0.03)	0.06 (-0.01, 0.13)
hsa-miR-125a-5p	-0.04 (-0.15, 0.07)	-0.04 (-0.13, 0.05)	-0.06 (-0.15, 0.03)	-0.04 (-0.14, 0.07)
hsa-miR-125b-5p	-0.02 (-0.11, 0.07)	-0.01 (-0.09, 0.07)	-0.04 (-0.11, 0.04)	-0.01 (-0.10, 0.07)
hsa-miR-126-3p	-0.03 (-0.11, 0.05)	-0.01 (-0.07, 0.06)	-0.05 (-0.11, 0.02)	0.00 (-0.07, 0.08)
hsa-miR-128	-0.06 (-0.16, 0.05)	-0.06 (-0.15, 0.02)	-0.05 (-0.13, 0.04)	-0.06 (-0.16, 0.03)
hsa-miR-142-3p	-0.01 (-0.13, 0.12)	0.05 (-0.05, 0.16)	-0.02 (-0.13, 0.08)	0.06 (-0.06, 0.17)
hsa-miR-143-3p	0.07 (-0.02, 0.16)	0.06 (-0.01, 0.13)	0.04 (-0.04, 0.11)	0.06 (-0.02, 0.14)
hsa-miR-155-5p	-0.11 (-0.30, 0.07)	-0.12 (-0.27, 0.03)	-0.10 (-0.25, 0.05)	-0.14 (-0.31, 0.03)
hsa-miR-15a-5p	0.01 (-0.11, 0.13)	0.07 (-0.04, 0.17)	0.00 (-0.10, 0.11)	0.07 (-0.04, 0.19)
hsa-miR-15b-5p	0.00 (-0.11, 0.12)	0.03 (-0.07, 0.12)	-0.04 (-0.14, 0.05)	0.03 (-0.07, 0.14)
hsa-miR-16-5p	0.00 (-0.09, 0.10)	0.04 (-0.04, 0.12)	-0.04 (-0.12, 0.04)	0.05 (-0.04, 0.14)
hsa-miR-17-5p	-0.01 (-0.08, 0.07)	0.03 (-0.03, 0.09)	-0.02 (-0.08, 0.04)	0.04 (-0.03, 0.10)
hsa-miR-185	-0.06 (-0.16, 0.05)	0.02 (-0.07, 0.10)	-0.10 (-0.18, -0.01)*	0.02 (-0.07, 0.11)
hsa-miR-18a-5p	-0.02 (-0.13, 0.08)	0.02 (-0.07, 0.10)	-0.03 (-0.11, 0.06)	0.03 (-0.06, 0.13)
hsa-miR-195-5p	0.02 (-0.23, 0.27)	0.09 (-0.11, 0.30)	-0.04 (-0.24, 0.17)	0.11 (-0.12, 0.34)
hsa-miR-19a-3p	0.04 (-0.06, 0.15)	0.08 (-0.01, 0.17)	0.00 (-0.09, 0.08)	0.09 (0.00, 0.19)
hsa-miR-200c-3p	-0.05 (-0.15, 0.06)	-0.05 (-0.14, 0.03)	-0.05 (-0.14, 0.03)	-0.06 (-0.16, 0.04)
hsa-miR-20a-5p	-0.01 (-0.08, 0.07)	0.02 (-0.05, 0.08)	-0.02 (-0.08, 0.05)	0.03 (-0.04, 0.10)
hsa-miR-21-5p	-0.01 (-0.14, 0.11)	-0.05 (-0.15, 0.06)	-0.04 (-0.14, 0.07)	-0.04 (-0.16, 0.07)
hsa-miR-222-3p	0.01 (-0.06, 0.07)	0.01 (-0.05, 0.06)	-0.02 (-0.08, 0.03)	0.01 (-0.05, 0.07)
hsa-miR-25-3p	-0.02 (-0.14, 0.09)	0.01 (-0.09, 0.10)	-0.06 (-0.15, 0.04)	0.01 (-0.10, 0.11)
hsa-miR-26b-5p	-0.03 (-0.15, 0.09)	-0.03 (-0.13, 0.07)	-0.04 (-0.14, 0.06)	-0.03 (-0.14, 0.09)
hsa-miR-29c-3p	-0.04 (-0.11, 0.04)	-0.02 (-0.09, 0.04)	-0.03 (-0.09, 0.04)	-0.03 (-0.10, 0.04)
hsa-miR-30a-5p	-0.03 (-0.11, 0.05)	0.00 (-0.07, 0.07)	-0.05 (-0.11, 0.02)	0.00 (-0.07, 0.08)
hsa-miR-30c-5p	-0.01 (-0.10, 0.09)	-0.01 (-0.09, 0.06)	-0.03 (-0.10, 0.05)	-0.02 (-0.10, 0.07)
hsa-miR-92a-3p	-0.02 (-0.09, 0.05)	0.00 (-0.05, 0.06)	-0.04 (-0.10, 0.01)	0.00 (-0.06, 0.07)
hsa-miR-93-5p	-0.03 (-0.11, 0.05)	0.00 (-0.07, 0.07)	-0.03 (-0.10, 0.04)	0.01 (-0.07, 0.09)

DEHPm=DEHP metabolites, LMW=Low Molecular Weight Phthalates, HMW=High Molecular Weight Phthalates, CI=Confidence Interval.

*$p<0.05$, adjusting for maternal age, maternal ethnicity, and self-reported maternal smoking (Yes/No), and infant-sex

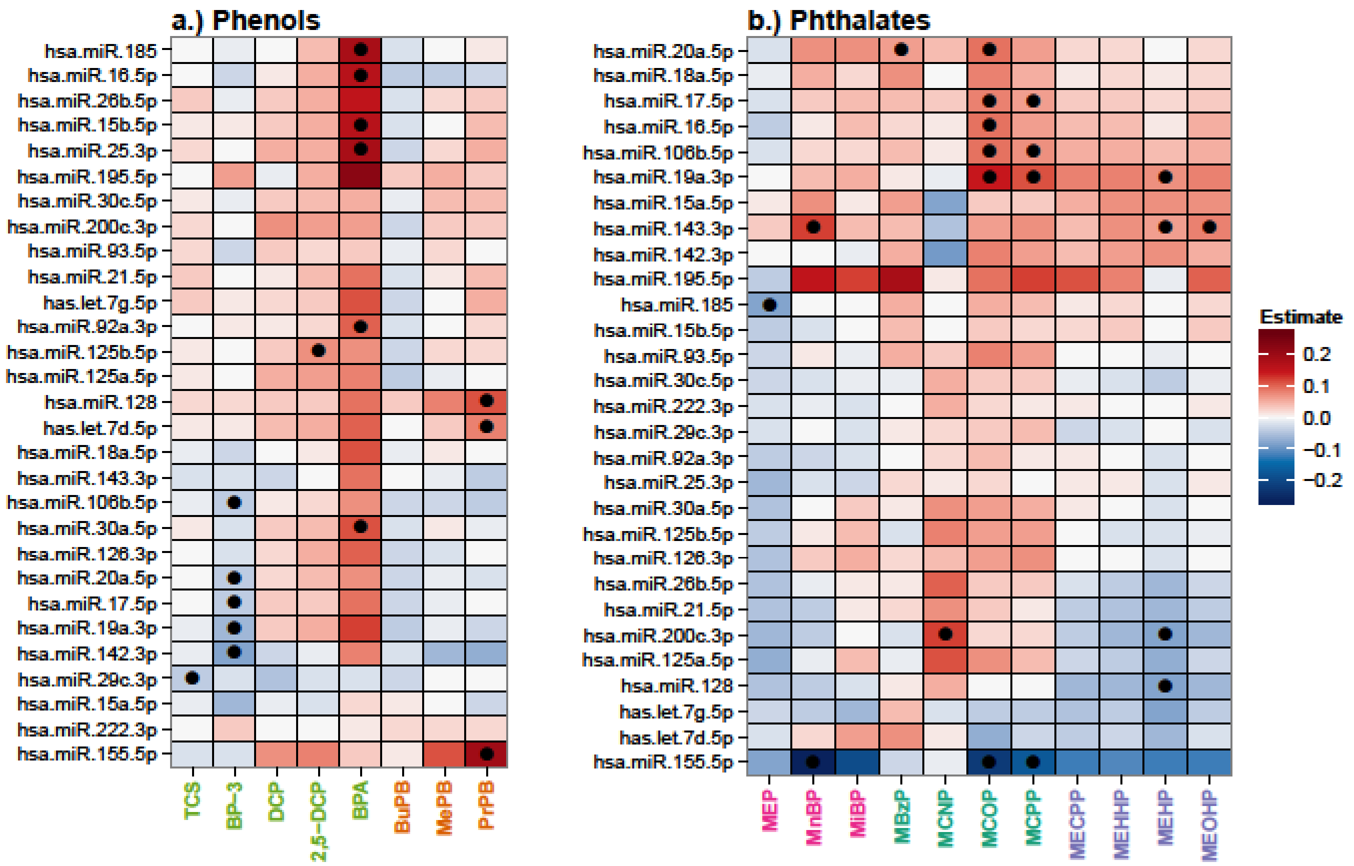

Figure S5. Association between miRNA Expression and each EDC Metabolite Independently. Each cell corresponds to a test for the association between an individual metabolite (columns) and the expression of a specific miRNA (rows). The cell color corresponds to the change in ΔCt miRNA expression with a one log(mol/L) increase in metabolite adjusting for maternal age, maternal ethnicity, and self-reported maternal smoking (Yes/No), and infant-sex; dots indicate $p<0.05$ for the association between metabolite and EDC in the multivariable model; green=non-paraben phenol, orange=paraben; pink=LMW phthalate; turquoise=HMW non-DEHP phthalate; purple=HMW DEHP phthalate.

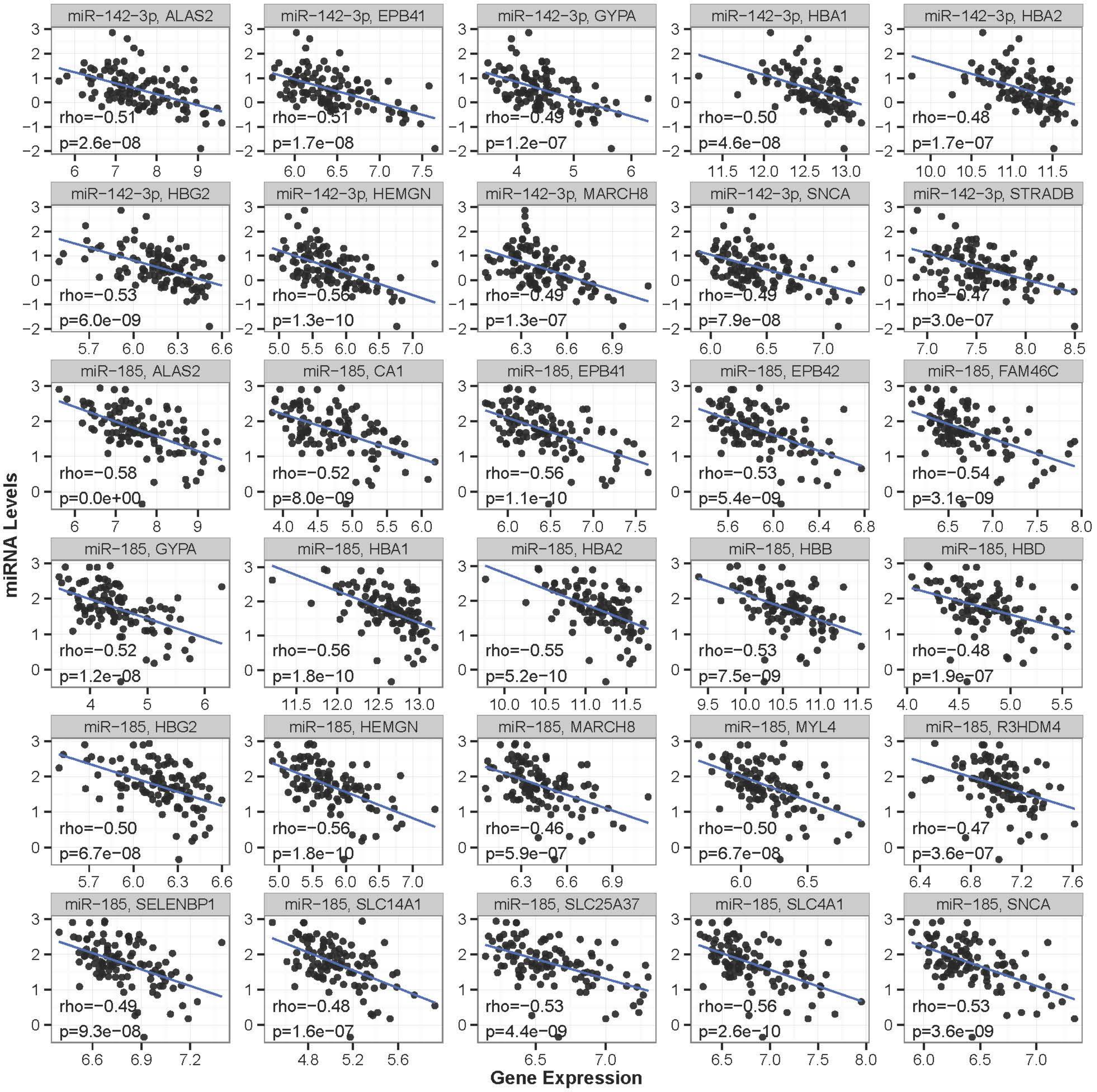

Figure S6. MicroRNA and gene expression levels among the subset of significant pairwise Spearman correlations identified among a subset of samples run on Affymetrix GeneChip® Human Gene 2.0 ST Array after adjusting for multiple testing.

Table S5. Correlations between birth outcomes and miRNAs significant among EDC profiles.

miRNA	Gestational Age (wks) Coefficient (CI)	Birth Weight (g) Coefficient (CI)	Birth Length (cm) Coefficient (CI)
hsa-miR-142-3p	-0.12 (-0.36, 0.12)	18.17 (-66.66, 103.00)	-0.04 (-0.49, 0.41)
hsa-miR-15a-5p	-0.14 (-0.38, 0.10)	-0.93 (-87.91, 86.05)	-0.23 (-0.68, 0.22)
hsa-miR-185	-0.03 (-0.32, 0.26)	1.33 (-99.86, 102.52)	0.14 (-0.39, 0.67)

CI=Confidence Interval
